# Implementation of Digital Awareness Strategies to Engage Patients and Providers in a Lung Cancer Screening Program: Retrospective Study

**DOI:** 10.2196/jmir.8932

**Published:** 2018-02-15

**Authors:** Dana L Jessup, McKinley Glover IV, Dania Daye, Lynda Banzi, Philip Jones, Garry Choy, Jo-Anne O Shepard, Efrén J Flores

**Affiliations:** ^1^ Department of Radiology Massachusetts General Hospital Boston, MA United States; ^2^ Massachusetts General Physicians Organization Boston, MA United States

**Keywords:** lung neoplasms, diagnostic imaging, social media, patient participation, search engine

## Abstract

**Background:**

Lung cancer is the leading cause of cancer-related deaths in the United States. Despite mandated insurance coverage for eligible patients, lung cancer screening rates remain low. Digital platforms, including social media, provide a potentially valuable tool to enhance health promotion and patient engagement related to lung cancer screening (LCS).

**Objective:**

The aim was to assess the effectiveness of LCS digital awareness campaigns on utilization of low-dose computed tomography (LDCT) and visits to institutional online educational content.

**Methods:**

A pay-per-click campaign utilizing Google and Facebook targeted adults aged 55 years and older and caregivers aged 18 years and older (eg, spouses, adult children) with LCS content during a 20-week intervention period from May to September 2016. A concurrent pay-per-click campaign using LinkedIn and Twitter targeted health care providers with LCS content. Geographic target radius was within 60 miles of an academic medical center. Social media data included aggregate demographics and click-through rates (CTRs). Primary outcome measures were visits to institutional Web pages and scheduled LDCT exams. Study period was 20 weeks before, during, and after the digital awareness campaigns.

**Results:**

Weekly visits to the institutional LCS Web pages were significantly higher during the digital awareness campaigns compared to the 20-week period prior to implementation (mean 823.9, SD 905.8 vs mean 51, SD 22.3, *P*=.001). The patient digital awareness campaign surpassed industry standard CTRs on Google (5.85%, 1108/18,955 vs 1.8%) and Facebook (2.59%, 47,750/1,846,070 vs 0.8%). The provider digital awareness campaign surpassed industry standard CTR on LinkedIn (1.1%, 630/57,079 vs 0.3%) but not Twitter (0.19%, 1139/587,133 vs 0.25%). Mean scheduled LDCT exam volumes per week before, during, and after the digital awareness campaigns were 17.4 (SD 7.5), 20.4 (SD 5.4), and 26.2 (SD 6.4), respectively, with the difference between the mean number of scheduled exams after the digital awareness campaigns and the number of exams scheduled before and after the digital awareness campaigns being statistically significant (*P*<.001).

**Conclusions:**

Implementation of the LCS digital awareness campaigns was associated with increased visits to institutional educational Web pages and scheduled LDCT exams. Digital platforms are an important tool to enhance health promotion activities and engagement with patients and providers.

## Introduction

Lung cancer is the leading cause of cancer-related deaths in the United States, with more than 157,000 deaths per year [1]. The National Lung Cancer Screening Trial found that lung cancer screening (LCS) with low-dose computed tomography (LDCT) resulted in a decrease in lung cancer mortality by 20% when screening high-risk patients [2]. As a result, the US Preventive Services Task Force issued a Grade B recommendation for annual screening of high-risk adults age 55 to 80 years for lung cancer, with LDCT being the only recommended screening test [3].

Section 2713 of the Affordable Care Act mandates that private insurance companies cover screening examinations with A or B recommendations from the US Preventive Services Task Force [4]. Additionally, the Centers for Medicare and Medicaid Services issued a coverage directive in February 2015 that LCS counseling and shared decision-making visits are covered services along with LDCT screening for eligible beneficiaries [5]. However, despite mandated coverage of LDCT for eligible patients by public and private payors, reported screening rates of eligible patients have remained under 4% as of 2015 [6].

There are many potential reasons for relatively low rates of LCS among eligible patients [7]. Patients and providers may not be aware of the availability and importance of this relatively new screening test [8,9]. Additional potential contributors to low LCS rates include cost concerns, radiation dose concerns, physician and/or patient ambivalence about the mortality benefits of screening, social stigma associated with smoking and lung cancer among patients, lack of physician knowledge about screening eligibility, and uncertainty regarding insurance coverage and reimbursement [10-13].

The Internet and digital platforms, including social media, provide unique tools for health care organizations and providers to engage in public health and health promotion initiatives [14]. The Internet has become a dominant source of health information: 72% of adult Internet users go online for health information, with the majority of searches initiated through search engines [15]. Studies have also demonstrated the value of social media in emergency preparedness, epidemiology, health education, and patient engagement [16-20]. However, social media has the potential to negatively impact health behaviors [14]. Nevertheless, the power of social media as a tool to foster engagement between health care providers and patients appears to be growing [14].

Shared decision making using one or more decision aids is required for coverage by the Centers for Medicare and Medicaid Services and strongly emphasized in clinical practice. Thus, education and outreach to both patients and providers are critical to improving screening rates among eligible patients. To that end, social media and search engine outreach present a unique opportunity to promote awareness of a screening examination that provides a clear mortality benefit.

The purpose of this study was to determine if a patient- and provider-focused LCS digital awareness campaign was associated with (1) utilization of LDCT and (2) engagement with online educational content on LCS and LDCT.

## Methods

This single-institution, retrospective study was exempt from the Institutional Review Board. A 20-week pay-per-click campaign was developed to target two specific populations within a 60-mile radius of a large quaternary medical center and two affiliated off-campus imaging sites: (1) patients and caregivers and (2) health care providers. Primary outcome was visits to institutional LCS Web pages. Secondary outcome was utilization of LDCT at the academic medical center. Utilization is defined as exams that were scheduled within the precampaign, campaign, and postcampaign 20-week periods and subsequently completed.

### Patient Awareness Campaign

Facebook (Menlo Park, CA, USA) and Google (Mountainview, CA, USA) were selected as the digital platforms for the patient awareness campaign due to their prevalent use among adult Internet users. Information about LCS for patients and caregivers, including eligibility, insurance coverage and the benefit of early detection, appeared on Facebook and Google search from May 12 to September 30, 2016. Using the options that these platforms make available to directly reach specific groups of people, the patient awareness campaign hypertargeted the following: current and former smokers aged 55 years and older, females aged 55 years and older, patients and employees of the academic medical center (individuals 18 years and older), and caregivers (ie, individuals aged 18 years and older).

**Figure 1:**
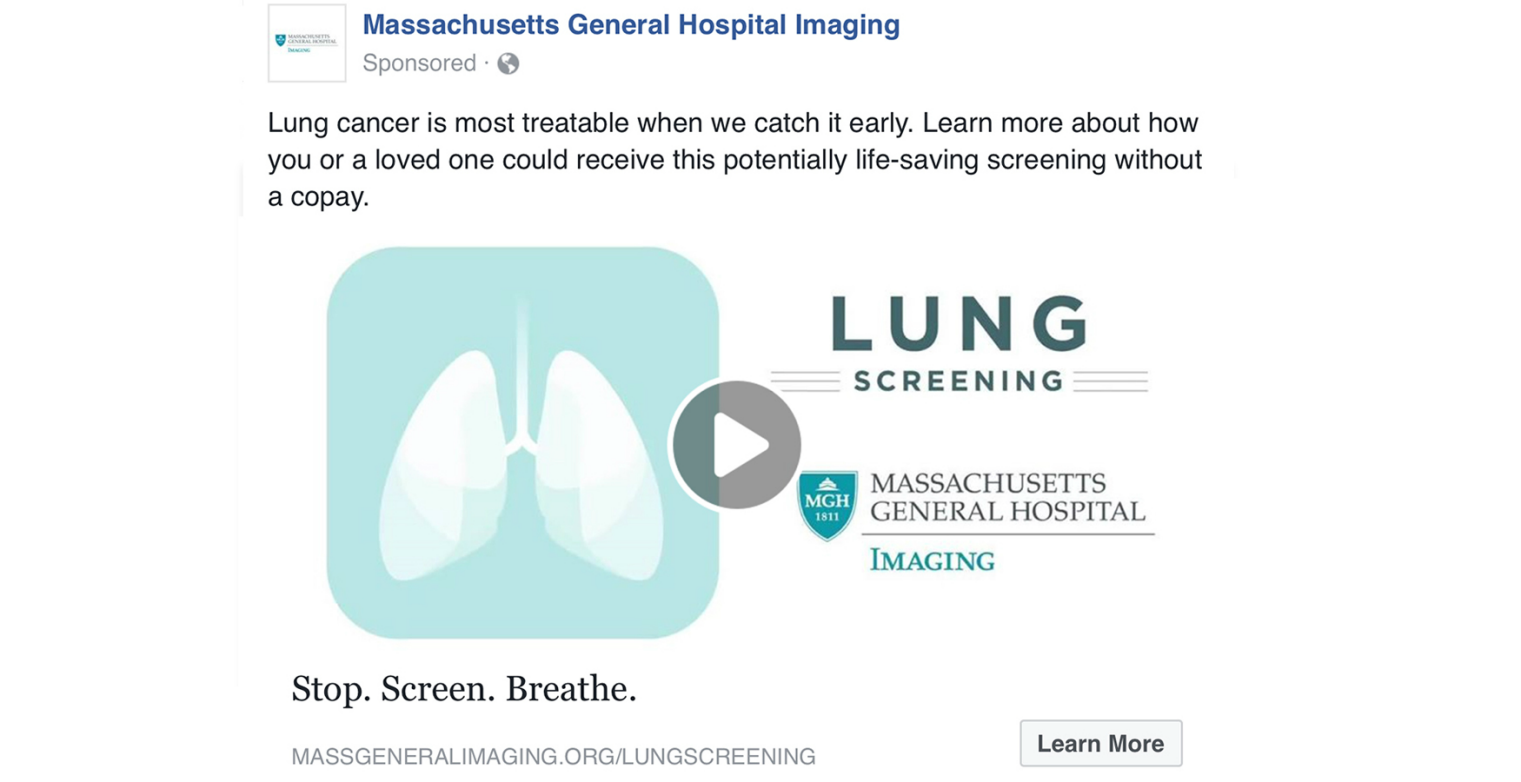
Patient-focused lung cancer screening content on Facebook included carousels, videos, graphics interchange formats, and static images.

Content on Facebook took several different forms including rotating sets of images (ie, carousels), two unique 30-second videos on the risks of smoking and screening eligibility, an animated graphics interchange format (GIF) depicting a growing lung nodule, and a static display of images including computed tomography (CT) technologists and CT scanners (Figure 1).

Content on Google search was primarily text and grouped by categories including signs and symptoms, smoking and lung cancer, and LCS eligibility. Content appeared when users in the specified geographic location searched designated keywords. Messages included “Don’t wait for symptoms,” “Lung screening saves lives,” and “Learn about screening eligibility.” Some content also addressed LDCT’s lower dose of radiation and stressed that treatment may be more effective with early detection.

All patient awareness content included links to one of two institutional Web pages: (1) general information about LCS and LDCT and (2) frequently asked questions (FAQ) tailored specifically for patients [21,22].

### Provider Awareness Campaign

LinkedIn (Mountainview, CA, USA) and Twitter (San Francisco, CA, USA) were selected to deliver provider-focused content from June 21 to September 30, 2016. The following health care provider characteristics were hypertargeted: primary care specialties (eg, family medicine, internal medicine) and licensed providers (eg, MD, NP, PA) who may see patients independently as a part of their practice.

**Figure 2:**
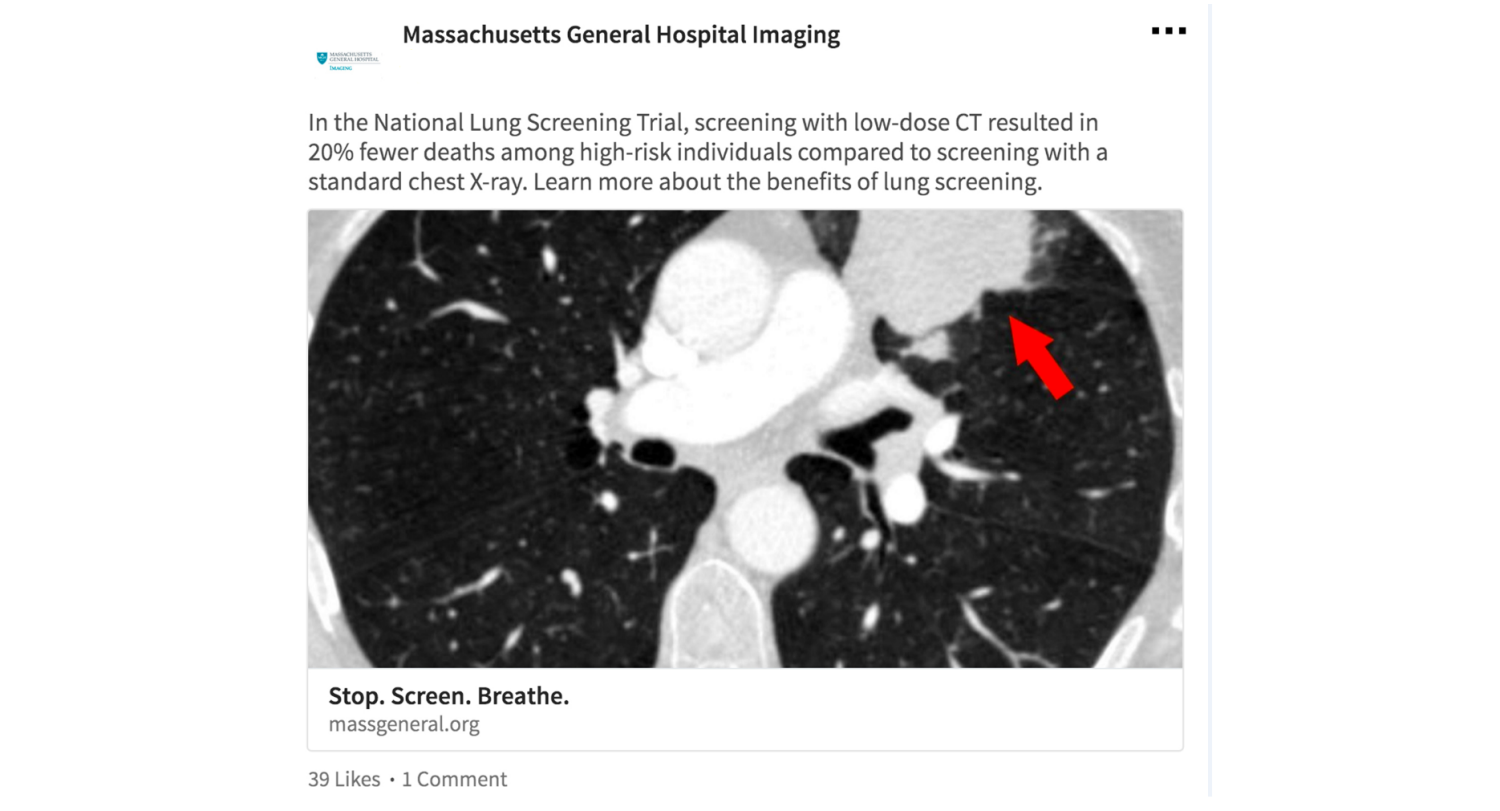
Provider-focused lung cancer screening content on LinkedIn focused on eligibility and mortality benefit.

Content on LinkedIn focused on LCS mortality benefit and eligibility criteria by referencing high-risk patients and identifying specific requirements for eligibility such as number of pack-years smoked (Figure 2). Content also addressed the value of a CT scan compared to a standard chest radiograph. In addition, providers were prompted to consider LCS counseling during a shared decision-making visit with high-risk patients.

**Figure 3:**
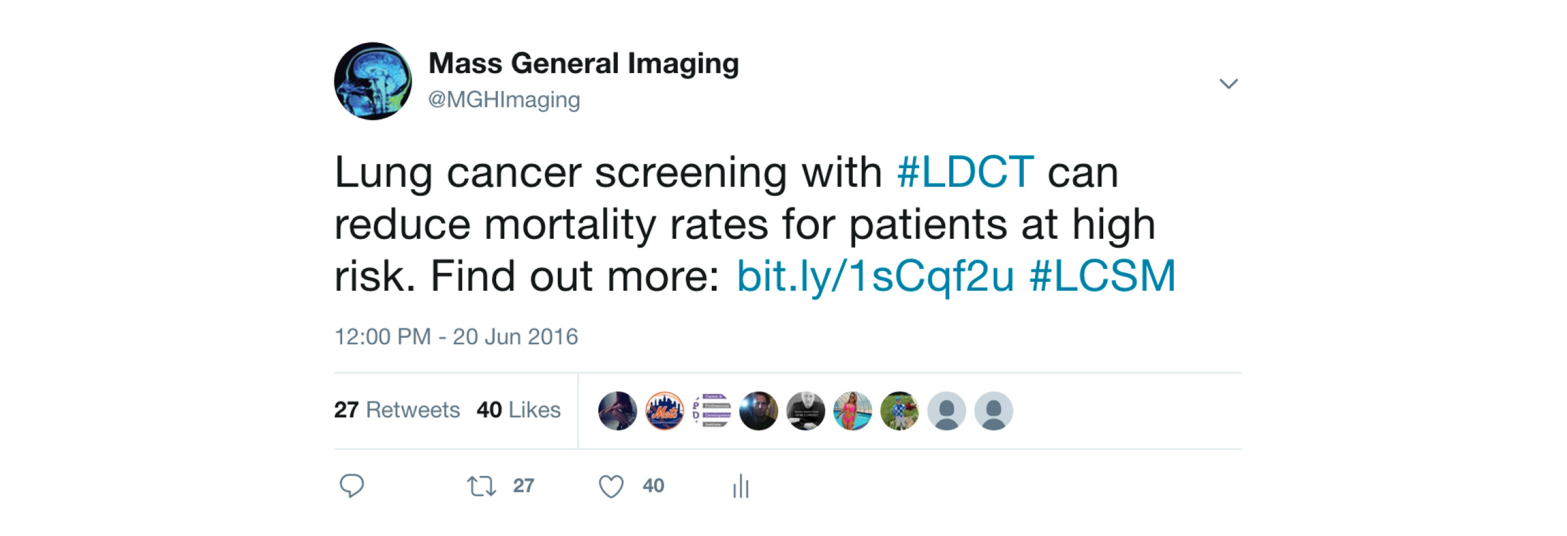
Provider-focused lung cancer screening content on Twitter focused on eligibility and mortality benefit.

Content on Twitter targeted a broad group of health care professionals and patient advocacy organizations, highlighting which patients may be eligible for LDCT without copay and the mortality benefit of LCS with LDCT. In some cases, content posed a question (eg, “Do you know someone who might be eligible?”) or featured an animated GIF depicting a growing lung nodule. Targeted keywords included “smoking cessation,” “lung cancer social media,” and “lung health” (Figure 3).

All provider-focused content included links to one of two institutional Web pages: (1) general information about LCS and LDCT and (2) FAQ tailored specifically for physicians [21,23].

### Data Collection

All digital platforms provided aggregate data on users’ geographic location (eg, by state, by municipality). Additionally, Twitter and Facebook provided aggregate data on age and gender, and LinkedIn provided aggregate data on job title (ie, provider type). Metrics were collected including “impressions” (ie, the number of times content was served on a platform) and “clicks” (ie, the number of times users clicked anywhere on the content). All platforms also provided information on click-through rates (CTRs) (ie, clicks/impressions).

Visits to institutional LCS Web pages as a result of the campaign were measured using Google Analytics Universal version and included the number and date of page views (ie, total number of pages viewed; includes repeated views of a single page) and "sessions" (ie, a period of time during which a user is actively engaged in a website; can include multiple page views). Data on LDCT examinations were obtained from the electronic medical record (Epic Hyperspace 2015 IU RA1836 Version 82.2.16, Verona, WI, USA), including the date the examinations were scheduled and performed.

### Statistical Analysis

The number of total scheduled exams per week was calculated for each week during the precampaign, campaign, and postcampaign 20-week periods. The mean number of visits to the patient FAQ Web page, physician FAQ Web page, and the general LCS information Web page before, during, and after the digital awareness campaigns were computed. Results are reported separately for each of the three Web pages.

Similarly, the mean number of exams scheduled before, during, and after the campaign were also calculated. Scheduled exams included in the analysis were subsequently performed. A one-way analysis of variance (ANOVA) was conducted to determine any statistically significant differences between the means of the assessed variable before, during, and after the digital awareness campaigns. Pairwise subgroup analysis was performed using a two-sided *t* test to assess for statistically significant differences between the mean number of scheduled exams at any two campaign periods. Bonferroni-adjusted *P* values were used to correct for multiple comparisons. In the subgroup analysis, a *P* value less than .016 was considered to be statistically significant to account for the three assessed subgroups (Bonferroni correction). Otherwise, a *P* value less than .05 was considered statistically significant in all other analyses. Statistical analyses were performed with Stata 14 (College Station, TX, USA).

## Results

### Patient Awareness Campaign

Facebook generated 1,846,070 impressions with a CTR of 2.59% (47,750/1,846,070), which was higher than the reported health care industry average of 0.8% [24]. The highest CTR was among individuals aged 18 to 24 years (3.21%, 13,238/411,200). Facebook metrics by gender and age are presented in Table 1. Google search generated 18,955 impressions, with a CTR of 5.85% (1108/18,955) compared to a health and medical industry average of 1.8% [25]. Highest CTRs resulted from content that referenced signs of lung cancer (17.09%, 335/1960) and the benefits of early detection (10.4%, 22/211).

### Provider Awareness Campaign

LinkedIn generated 57,079 impressions with a CTR of 1.10% (630/57,079) compared to an overall industry average of 0.3% [26]. The 630 clicks came from physicians (10.2%, 64/630), registered nurses (9.5%, 60/630), nurse practitioners (5.7%, 36/630), nurses (2.4%, 15/630), physician assistants (2.2%, 14/630), clinical specialists (2.1%, 13/630), and other job title categories (67.9%, 428/630). Content with the highest CTR contained statistics about patients most at risk of lung cancer and the mortality benefit of LDCT. Twitter generated 587,133 impressions with a CTR of 0.19% (1139/587,133) compared to an overall industry average of 0.25% (oral communication, H Justin, sales manager, Twitter, June 2016).

Table 2 presents campaign performance per platform as defined by (1) comparison with industry standard CTRs and (2) resulting online sessions that included institutional Web pages on LCS.

### Visits to Institutional Web Pages

Visits to institutional Web pages on LCS are presented in Table 3. The mean weekly visits for the institutional LCS general Web page before, during, and after the campaign were 51.0 (SD 22.3), 823.9 (SD 905.8), and 438.8 (SD 1094.5), respectively (*P*=.03). The mean weekly visits to the institutional patient FAQ webpage before, during, and after the campaign were 11.5 (SD 6.9), 535.3 (SD 484.9), and 131.2 (SD 283.7), respectively (*P*<.001). The mean weekly visits to the institutional provider FAQ webpage before, during, and after the campaign were 5.2 (SD 2.6), 90.8 (SD 92.7), and 28.6 (SD 45.5), respectively (*P*<.001).

### Low-Dose Computed Tomography Utilization

**Figure 4:**
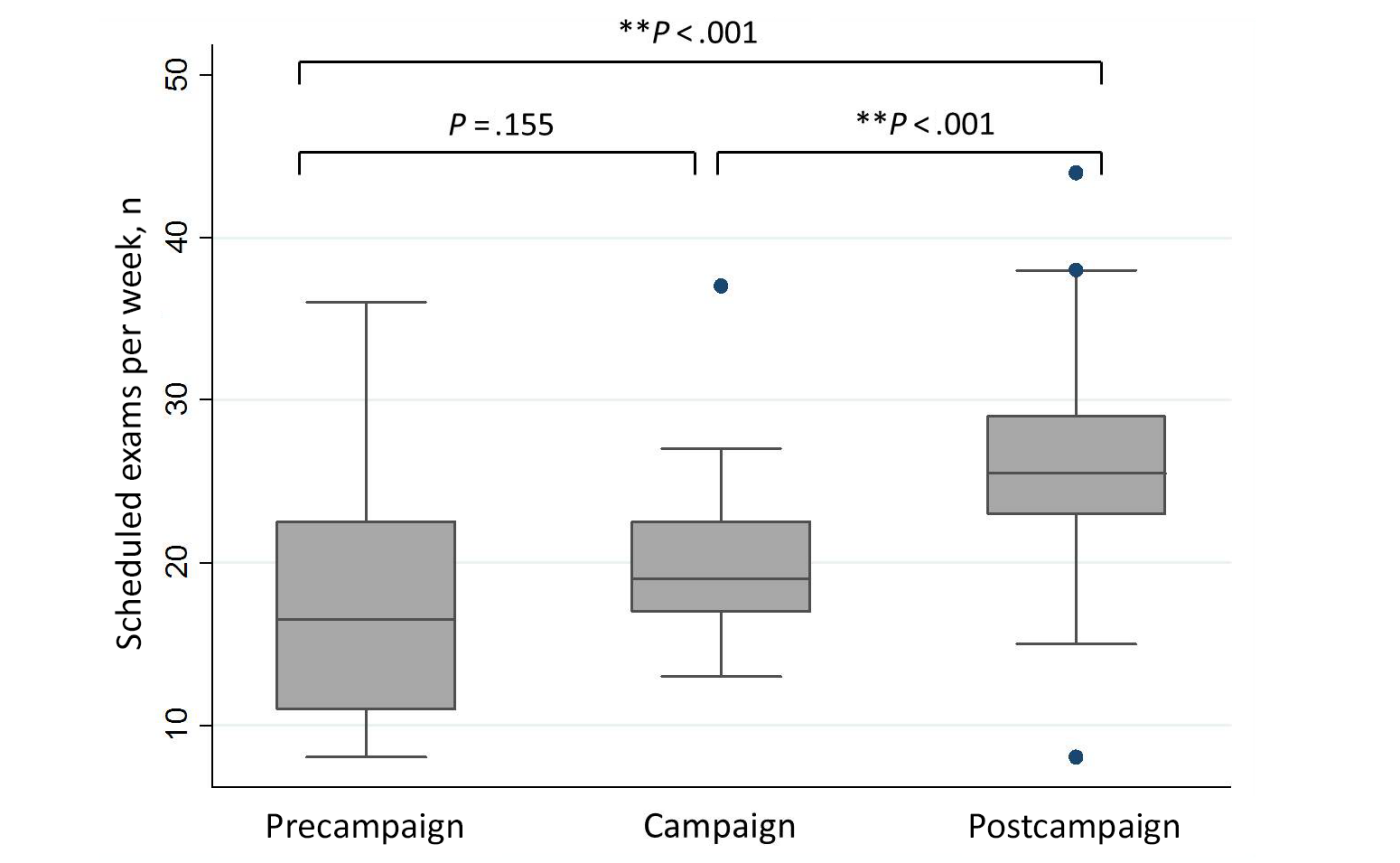
The differences between the mean number of weekly scheduled exams before, during, and after the campaigns. The boxes represent the upper and lower quartiles of the dataset, the whiskers extend to the highest and lowest observations in the dataset that are one and a half times the interquartile range, and the dots represent the outliers observed in the dataset.

During the 20 weeks before the study period, 349 LDCT exams were scheduled, resulting in a mean 17.4 (SD 7.5) exams per week. During the 20-week study period, 415 LDCT exams were scheduled, resulting in a mean 20.4 (SD 5.4) exams per week. During the 20 weeks after the study period, 483 exams were scheduled, resulting in a mean 26.2 (SD 6.4) exams per week (Figure 4). All these exams were subsequently performed. ANOVA analysis revealed statistically significant differences between the means of the total number of weekly exams scheduled before, during, and after the digital awareness campaigns (*P*<.001). Pairwise subgroup analysis revealed statistically significant differences between the mean number of scheduled exams during and after the digital awareness campaigns and before and after the digital awareness campaigns (*P*<.001).

**Table 1:** Facebook metrics by gender and age.

Demographic			Impressions, n		Reach, n		Clicks, n		CTR, %		Sessions, n
**Age**											
	18-24	411,200		192,388		13,238		3.21		2658	
	25-54	557,542		273,412		12,879		2.30		6304	
	≥55	876,419		266,931		21,632		2.46		3264	
**Gender**											
	Male	797,492		319,848		23,042		2.89		3021	
	Female	1,028,935		406,128		24,320		2.36		4487	

**Table 2:** Campaign performance by digital platform. CTR: click-through rate.

Campaign			Impressions, n		Clicks, n		Campaign CTR, %		Industry standard CTR, %		Sessions, n
**Provider awareness**											
	Twitter	587,133		1139		0.19		0.25		927	
	LinkedIn	57,079		630		1.10		0.3		595	
**Patient awareness**											
	Facebook	1,846,070		47,750		2.59		0.8		13,341	
	Google	18,955		1108		5.85		1.8		1522	

**Table 3:** Mean weekly visits to institutional Web pages. FAQ: frequently asked questions; LCS: lung cancer screening.

Institutional Web page	Precampaign, mean (SD)	Campaign, mean (SD)	Postcampaign, mean (SD)	*P*
General LCS information	51.0 (22.3)	823.9 (905.8)	438.8 (1094.5)	.03
Physician FAQ	5.2 (2.6)	90.8 (92.7)	28.6 (45.5)	<.001
Patient FAQ	11.5 (6.9)	535.4 (484.9)	131.2 (283.7)	<.001

## Discussion

This single-institution study found that simultaneous digital awareness campaigns focused on both patients and providers were associated with an increase in visits to institutional Web pages on LCS and scheduled LDCTs. This study has several important implications, particularly for stakeholders in health promotion and population health management.

The digital awareness campaigns were associated with a significant increase in visits to institutional Web pages on LCS. This finding suggests the possibility that the campaign was successful in providing patients and providers with information on LCS and LDCT. Patient education, in particular for high-risk patients, is a fundamental component of the shared decision-making process for LCS [27]. Social media and search engine platforms provide an important vehicle to connect with potential patients who may not be integrated into the health care delivery system.

Secondly, the patient-focused digital awareness campaign surpassed reported health care industry standards for CTRs. There are several potential explanations for this finding. First, interest in LCS-related content may be higher than general industry standards for health care-related topics due to the increased interest in lung cancer when compared to the broader landscape of health care information disseminated on social media platforms. Alternatively, hypertargeting of the patient demographic groups may have contributed to the higher-than-industry-standard CTRs observed in this study. Interestingly, the highest CTRs observed were among individuals aged 18 to 24 years. This finding may be related to higher rates of social media utilization among younger individuals. However, in development of social media outreach efforts, identifying caregivers and family members that may have an influence over health care decisions has been shown to be an important aspect in patient engagement [28]. In the case of LCS, integrating younger individuals into discussions about their loved ones’ screening decision may also influence their own smoking behavior.

Within the provider-focused digital awareness campaign, LinkedIn surpassed industry standards for CTRs, whereas Twitter did not. At first glance, this finding may appear surprising, given that LinkedIn is primarily used for networking between professionals. However, social networking sites for professionals may have more robust targeting algorithms to reach providers, given the greater specificity in the job titles of their users. This may allow for enhanced ability to provide content to individuals who are more likely to be interested in LCS. Therefore, institutions that are considering provider education campaigns should consider utilization of such sites. Academic institutions may not be leveraging these sites fully, which may represent untapped potential to connect with health care providers within and outside of the organization [29].

Lastly, the number of scheduled LDCTs was significantly higher during and after the digital awareness campaigns when compared to the precampaign study period. Although this observational study does not allow for causality to be inferred, the findings suggest that digital awareness campaigns have the potential to not only provide education, but also influence behavior. Previous research has demonstrated that social media may be more cost-effective and have a broader reach than traditional media in recruiting patients eligible for LCS into research studies [30].

Future research may benefit from surveying patients undergoing LDCT to ascertain which outreach efforts may have influenced their decision to pursue LCS.

There are several limitations to this retrospective observational study. Importantly, the observational nature precludes determination that the digital awareness campaigns had a causal relationship with LDCT utilization. Although paid placements on social and search platforms clearly drove traffic to institutional Web pages, CTRs may have been above industry standards as a reflection of content branded to an academic medical center with significant brand equity in the targeted geographic area.

Additionally, the study did not control for other institutional, local, or national initiatives related to LCS, which may have influenced outcome measures. In particular, institutional initiatives during the 20 weeks after the digital awareness campaigns promoted the LCS general Web page as part of Lung Cancer Awareness Month activities, likely accounting for its relatively high mean daily visits during this period. Also, the number of shared decision-making visits was not readily accessible, which may be an additional proxy for the potential impact of education influencing patient and/or provider behavior. Further, the time required for obtaining a shared decision-making visit with a clinical provider could contribute to lead time bias in LDCT utilization.

Finally, demographic data from the digital platforms were provided in aggregate, which limits assessment of patient and provider demographics. Similarly, statistical significance of differences between the digital awareness campaign metrics and industry standards cannot be determined. Demographic data, including race, ethnicity, and socioeconomic status, of patients who received a LDCT before, during, or after the 20-week study period were not analyzed as part of this study.

Concurrent patient- and provider-focused digital awareness campaigns on LCS were associated with increased visits to online educational content and increases in the number of LDCT examinations. Digital platforms appear to be an important tool in health promotion and educational initiatives related to LCS with the potential to impact care-seeking behavior.

## Abbreviations

CT: computed tomography
CTR: click-through rate
FAQ: frequently asked questions
GIF: graphics interchange format
LCS: lung cancer screening
LDCT: low-dose computed tomography
